# Health, Happiness, and Gold

**Published:** 1864-12

**Authors:** 


(FROM THE "INDEPENDENT.")

### HEALTH, HAPPINESS, AND GOLD.

The decease of two prominent mercantile men, in the prime of life, in circumstances of ease, if not of opulence, and quite recently in the enjoyment of robust health, surrounded by every condition, in the business and family relations, which could render life desirable and promise a happy continuance of it, calls for some comment at our hands.

How is it that so many of our successful merchants are cut short in their earthly career, just at the period when it would seem that they had acquired all the requisites to make a deep mark upon the body social in which they lived? We recall two more instances, both partners in a large retailing dry-goods establishment in the city, the largest and probably the most successful one ever known here; they both died not long since, at the age of fifty, each in the possession of a large fortune, and, to all appearances, endowed with superior means of enjoying it.

Is it not too clearly manifested, in the premature deaths of these and numerous others in like circumstances, that there is something radically wrong in the habits and methods of life practiced by our mercantile men? Is there not too much tension of the nervous system, too much taxation of the brain?—in short, is there not entirely too much grasping for *rapid* accumulations? The latter is, undoubtedly, the cause of the other developments. Of what avail is it to acquire a large fortune, if one of the most probable consequences of it is the prostration of mental and physical power, and sheer inability to enjoy life in any form? How much better would it be if our business men, of superior capacity and resources, were to content themselves with aiming for a smaller fortune, and risking less to obtain it. These things are great or small relatively. Not many years ago, fifty thousand dollars would have been reckoned a competency; but what is now considered a generous fortune? How many well established merchants now contemplate retiring from active business with less than half-a-million, and how many expect ever to retire while they are making money?

We do not advocate ever retiring from *labor;* on the contrary, we know that constant employment is absolutely necessary for the happiness, and also for the health of every human being. We only mean to say that when a large fortune, or even a competency, has been acquired, that *then* the mind, strength, and energies of a business man, a part of the time at least, should change the current from self to the *world*, seeking the elevation and welfare of others less fortunate. At such a period the ordinary risks of business should be curtailed, so that the *brain* can be left more free for benevolent action.

A gentleman residing in the interior of our State, a farmer and president of a bank, called on us a few days since. He presented the vigorous appearance of a man in full health—his age might be fifty or thereabout. He had left New York city with what would be considered a very moderate competency, but which he thought sufficient, at the age of forty; and, to judge from appearances, he seems to possess everything that an intelligent and cultivated gentleman needs to make life enjoyable—namely, a family, a farm, a competency, and agreeable occupation. He is just now on the point of making his second European tour. In conversation upon the recent suicide of Mr. Leupp, he remarked that he wondered that there were not more cases of the sort, for that the business men he saw everywhere in this city appeared to him like a crowd of lunatics. And yet he is a business man, needing healthful excitement, but finding it in rational pursuits, study, and proper physical and mental exercise. Oh, when will our business men learn wisdom, and understand the laws of their physical being! What an example they are exhibiting, in multitudes of instances, of a blind idolatry of wealth and power, regardless of consequences! How many more cases of premature death shall we have to record from heart disease, from paralysis, from sudden insanity, caused by undue and phrensied devotion to what is called business? Friends, neighbors, fellow-toilers for earthly dross, let us pause and consider if we are not also in this category, and ask ourselves if we are not paying too dearly for our toys, even though they assume the form and dimensions of a colossal *Golden Calf*.

---

(FROM THE "INDEPENDENT.")

